# Understanding consumers’ intentions to purchase smart clothing using PLS-SEM and fsQCA

**DOI:** 10.1371/journal.pone.0291870

**Published:** 2023-09-19

**Authors:** Shucong Chen, Jing Ye

**Affiliations:** 1 Department of Fashion and Accessory Design, College of Design, Jiaxing University, Jiaxing, China; 2 Department of Fashion Design and Engineering, College of Design, Jiaxing University, Jiaxing, China; Universiti Tenaga Nasional, MALAYSIA

## Abstract

With the advancement of artificial intelligence (AI) and the Internet of Things (IoT), smart clothing, which has enormous growth potential, has developed to suit consumers’ individualized demands in various areas. This paper aims to construct a model that integrates that technology acceptance model (TAM) and functionality-expressiveness-aesthetics (FEA) model to explore the key factors influencing consumers’ smart clothing purchase intentions (PIs). Partial least squares structural equation modeling (PLS-SEM) was employed to analyze the data, complemented by fuzzy-set qualitative comparative analysis (fsQCA). The PLS-SEM results identified that the characteristics of functionality (FUN), expressiveness (EXP), and aesthetics (AES) positively and significantly affect perceived ease of use (PEOU), and only EXP affects perceived usefulness (PU). PU and PEOU positively impact consumers’ attitudes (ATTs). Subsequently, PU and consumers’ ATTs positively influence PIs. fsQCA revealed the nonlinear and complex interaction effects of the factors influencing consumers’ smart clothing purchase behaviors and uncovered five necessary and six sufficient conditions for consumers’ PIs. This paper furthers theoretical understanding by integrating the FEA model into the TAM. Additionally, on a practical level, it provides significant insights into consumers’ intentions to purchase smart clothing. These findings serve as valuable tools for corporations and designers in strategizing the design and promotion of smart clothing. The results validate theoretical conceptions about smart clothing PIs and provide useful insights and marketing suggestions for smart clothing implementation and development. Moreover, this study is the first to explain smart clothing PIs using symmetric (PLS-SEM) and asymmetric (fsQCA) methods.

## Introduction

In the rapidly advancing fields of artificial intelligence (AI) and the Internet of Things (IoT), the wearable device market has experienced rapid growth, offering a variety of products to fulfill the needs and desires of interested consumers [1]. According to MarketsandMarkets (2019), the wearable technology market is projected to expand at a compound annual growth rate (CAGR) of 11.2% from 2016 to reach $56.8 billion by 2025. Wearable devices can be found in a variety of industrial areas, including the fashion industry, where they are integrated into clothing to enhance its value with electronic components. A prominent subcategory of this technology is smart clothing, which integrates sensors and information technology into garments [2]. Recently, smart clothing has seen increased growth. According to MarketsandMarkets, the smart clothing global market is expected to grow from $1.6 billion in 2019 to $5.3 billion in 2024, for a CAGR of 26.2%. Additionally, Gartner (2019) predicted a surge in smart clothing shipments from 5.65 million in 2018 to 19.9 million in 2022.

As a form of wearable device, smart clothing is next-generation clothing or accessories empowered by information or electronic technology and wearable devices to offer the dual functions of perception and feedback [2]. Smart clothing not only detects changes in the external or internal environment but also responds to these changes through a feedback mechanism [3]. With advancements in wearable devices and clothing as essential equipment in daily life, smart clothing is receiving increasing attention from various perspectives. Some researchers have investigated innovative technologies in related sectors, including electronic information [4], sensors [5], and novel smart textiles [6]. Moreover, various researchers have paid attention to the applications of smart clothing, including smart healthcare [7], baby and elderly monitoring [8, 9], sports and wellness [10, 11], industry, defense and public safety [12, 13], and environmental interactions [14]. Moreover, numerous researchers have focused on the design of smart clothing. For instance, Li et al. [15] defined design principles for smart clothing, including intelligent module design and carrier design. Imbesi and Scataglini [16] proposed a user-centered framework for designing smart clothing for older adults. Chen et al. [17] introduced smart clothing design features, key technologies, and practical implementation methodologies. Although the prospects and functions of smart clothing are promising, most researchers have focused on smart clothing technology development and design, while few studies have researched consumer needs, attitudes (ATTs), and purchase intentions (PIs) related to smart clothing.

China, which is among the world’s fastest-growing regions for smart clothing, anticipates total market revenue of $17.5 billion between 2023 and 2029. The Chinese government has increased investment in research and development, talent training, and industry chain construction to actively promote the development of the smart apparel industry. Additionally, the government has issued a series of policy measures to support the smart clothing industry. Under these circumstances, the number of smart clothing consumers in China is increasing. Although this region has made significant progress in the development of smart clothing, it still encounters important development challenges, and the adoption of smart clothing remains in a relatively early stage. Furthermore, to the best of authors’ knowledge, research on consumer needs and PIs in emerging regions such as China is limited.

Previous studies have examined smart clothing PIs from various perspectives. Mahmood and Lee [18] explored how social influence (SI) and performance expectancy (PE) impact elderly users’ adoption of health-monitoring smart clothing. Nam and Lee [19] introduced an extended wearable acceptability range (WEAR) scale into the smart clothing field to investigate consumers’ social acceptance of smart clothing. Ju and Lee [2] found that perceived risks and unavailability lead to resistance to innovation in smart clothing. Park and Noh [20] investigated the effect of price sensitivity and innovativeness on consumers’ smart clothing behavioral intentions (BIs). Most related works have focused on consumers’ social, innovation, and technology acceptance. Moreover, most social acceptability (SA) constructs are adopted from the WEAR scale, while few studies have examined consumers’ needs to drive smart clothing purchases.

Lamb and Kallal [21] proposed the functionality-expressiveness-aesthetics (FEA) consumer needs model, which is widely utilized in identifying and evaluating target consumers’ clothing needs and is employed in designing any type of apparel. Lv et al. [22] explored the physiological, functional, aesthetic (AES) and psychological needs of elderly Chinese users of smart clothing. According to Li et al. [15], consumers not only prioritize the functionality (FUN) of smart clothing but also have heightened expectations regarding its appearance. Multiple Chinese studies using varied surveys have indicated that in the purchase of smart clothing, the needs are to improve uses’ health condition (HC) and increase their fashion sense, which also aligns with the FEA framework. Additionally, various studies have utilized the FEA model as an antecedent to investigate consumers’ needs for smart clothing, suggesting that the model constructs are feasible. Therefore, this work also incorporates the FEA model to explore the essential intrinsic attributes of smart clothing. Notably, the FEA model has yielded contrary conclusions across countries, and few studies have applied the FEA model to investigate Chinese consumers’ BIs. Therefore, this paper aims to examine the applicability of the FEA model in studying Chinese consumers’ behaviors toward smart clothing.

Various theoretical models, such as the theory of reasoned action (TRA) [23], the theory of planned behavior (TPB) [24, 25], the unified theory of acceptance and use of technology (UTAUT) [18], and the technology acceptance model (TAM) [26, 27], have been utilized to examine smart clothing PIs. The TAM, which is a well-known theoretical framework for understanding users’ motivations to accept new technology, has been widely applied in various studies related to smart clothing BIs. Although this framework has significant explanatory power, it may be too general to account for the specific factors of the phenomenon under investigation. Most TAM-based studies either incorporate additional variables to enhance overall their explanatory power or merge the TAM with other theories to solidify the theoretical foundation of the research model. Tsai et al. [28] extended the TAM by considering the impact of perceived prevalence, technology anxiety (TA), and resistance to change (RC) on patients’ wearable healthcare behavior. Additionally, few studies have examined the relationship between perceived ease of use (PEOU) and PIs in a smart clothing context. Moreover, several researchers have combined the FEA model with the TAM to investigate consumers’ smart clothing PIs, but the conclusions have varied across studies. Furthermore, most research has examined the relationships between AES attributes and consumers’ ATTs and BIs. Smart clothing should be designed to look good and contain various features that consumers feel are useful; additionally, the clothing should be easy to wear. However, few studies have examined the relationships among AES attributes and PU and PEOU. Therefore, this work incorporates consumers’ needs into the TAM to thoroughly understand how consumer needs influence BIs. To fill the gaps noted above, this study uses mediation analysis to explore the relationship between PEOU and PIs.

Methodologically, most previous studies on smart clothing PIs have employed symmetric approaches, such as covariance-based structural equation modeling (CB-SEM). Few studies have applied variance-based SEM (VB-SEM), commonly known as partial least squares structural equation modeling (PLS-SEM). In contrast to CB-SEM, PLS-SEM is based on a composite model [29] and offers more modeling flexibility with regard to modeling (e.g., formative and reflective measurement models) and data needs (e.g., smaller sample sizes and nonnormally distributed data) [30]. Few studies have employed VB-SEM to investigate smart clothing PIs. Additionally, the traditional symmetric technique focuses exclusively on the individual or net effects of antecedents and outcomes, overlooking the intricate configurations of variables.

To fill this gap, it is critical to assess the sufficiency and necessity of the factors under study in achieving the expected outcome [31]. This evaluation should occur from the vantage point of complexity theory and configurational models, thus making complexity theory an ideal theoretical framework for this research. As the most widely used asymmetric method, fuzzy-set qualitative comparative analysis (fsQCA), is often utilized to identify models that are both consistent and sufficient in predicting behavioral outcomes. Originally proposed by Ragin [32], fsQCA integrates qualitative and quantitative methodologies and can be applied to investigate how various causal condition combinations may result in the same outcome, using configuration analysis to explain complicated situations. With these considerations, the current study applies complexity theory in conjunction with fsQCA to comprehend the causal patterns underlying smart clothing PIs.

This paper explores the multifaceted influences on consumers’ intentions to purchase smart clothing. Drawing from a review of the relevant literature, an integrated theoretical model combining the FEA model and the TAM—both commonly used in smart clothing PI research—is developed. This analysis, based on an online survey of 225 Chinese consumers, employs both symmetric (PLS-SEM) and asymmetric (fsQCA) techniques to examine these influences. This study starts with an investigation of the individual effects of each antecedent using PLS-SEM, followed by mediation analysis to understand the relationship between PEOU and PIs. Additionally, to facilitate a more accurate comprehension of the complex reality associated with the diverse determinants and PIs, fsQCA is used for a more nuanced understanding of causal factor configurations. This approach elucidates the complexities of intention that cannot be fully explained by PLS-SEM alone. Insights gleaned from both PLS-SEM and fsQCA contribute perspectives and offer practical marketing suggestions to boost consumer’s purchase of smart clothing.

Based on the discussion above, the novelty of this paper can be summarized as follows. First, this study specifically focuses on Chinese consumers, addressing the growing market in China and filling the gap in research on consumer perspectives in this emerging region. Second, this research examines some understudied relationships, including the relationships among AES attributes and PU and PEOU, as well as the mediating effect of PU and ATTs on the relationship between PEOU and PIs. By exploring these relationships, this research contributes to advancing knowledge in the field and provides valuable insights for future research. Third, based on PLS-SEM analysis, this research examines the mediating role of PU and ATTs. Incorporating mediation analysis adds an additional layer of depth and strengthens the overall findings of the PLS-SEM analysis, offering valuable insights into the factors shaping consumers’ intentions toward smart clothing.

This work makes three core contributions. First, this research develops an integrated framework that combines the FEA consumer needs model with the TAM to explore smart clothing PIs in China’s burgeoning market. Second, this research enhances the theoretical understanding of the complexities involved in consumers’ PIs. Methodologically, this work innovatively employs an asymmetric technique (fsQCA) to supplement the traditional symmetric approach (PLS-SEM) frequently employed in previous studies. Symmetric methods predominantly focus on the individual and net effects of the determinants of smart clothing PIs. To the best of the authors’ knowledge, this study represents the first application of a method integrating PLS-SEM and fsQCA in the smart clothing context. Moreover, the hybrid approach can be extended to address other issues in the clothing area. Finally, on a managerial level, the insights derived from this study will help businesses and marketers develop effective strategies to convince consumers to purchase smart clothing.

The remainder of the paper is organized as follows: Section 2 presents the theoretical foundations, conceptual model, and hypotheses. The data and methodology are described in Section 3. Section 4 presents the PLS-SEM and fsQCA results. Section 5 provides the key findings and theoretical and managerial implications. Finally, Section 6 discusses the main conclusions and limitations of this study as well as potential future research.

## Literature review and hypothesis development

### Complexity theory

Complexity theory offers necessary tools for a significant shift in sociological practice, acknowledging that human behavior is partly shaped by dynamic social processes, but remains incompletely accounted for due to the distinct objective reality of humans. Complexity theory has been employed across disciplines to elucidate the heterogeneity, nonlinearity and dynamics inherent in complex systems [33]. Complexity theory has four basic principles: conjunction, equifinality, asymmetry, and causal asymmetry [34]. Conjunction implies that an outcome is the result of a combination of interdependent conditions, rather than being attributed to a singular cause [35]. Equifinality denotes that multiple diverse antecedent configurations can produce the same outcome [36]. Moreover, complexity theory recognizes the principle of asymmetry by allowing for the existence of contradictory instances. Consequently, the absence of antecedents that lead to high-scoring outcomes does not invariably result in low scores. Additionally, another core principle of complexity theory is causal asymmetry [35], where configurations resulting in high outcome scores and those leading to low scores are not mere inverses of each other. Causal asymmetry extends beyond linear theory, emphasizing the nonlinear relationships among the conditions that determine outcomes.

This study incorporates the principles of complexity theory by utilizing fsQCA, which merges qualitative comparative analysis with fuzzy-set principles [36]. The aptness of fsQCA for this study is supported by previous studies in similar nonlinear contexts [37]. This study acknowledges the necessity of incorporating an asymmetric method alongside symmetric analysis to fully capture the complexity of smart clothing PIs.

### Technology acceptance model (TAM)

In the current increasingly digital era, as new technologies continue to be developed and commercialized, several theoretical models have emerged to explore factors influencing technology adoption. In particular, the TAM, introduced by Davis [38], is one of the most important theories for assessing end-users’ adoption of new technology, which is derived from the TRA.

The TAM has various benefits with regard to assessing the factors that influence consumers’ intentions to utilize innovative technology. First, the TAM is a consistent measurement tool and reflects empirical rationality and simplicity [39]. Second, the TAM accounts for the majority of the variation in use intentions [40]. Third, the TAM has been used in several areas and increases the reliability and relevance of the questions in questionnaires by providing a variety of questions relevant to each factor [41]. Previous research has concluded that the TAM framework provides insights to better understand consumers’ acceptance of new technology-related applications by adding more external factors [42, 43]. Therefore, this study adopts the TAM as the main theoretical framework.

The TAM involves two key constructs, perceived usefulness (PU) and PEOU, both of which are influenced by external variables and affect users’ ATTs and behaviors with regard to accepting a new technology [38]. Some researchers have investigated the impact of PU and PEOU on the intention to use wearable technologies [27, 43]. The TAM incorporates ATTs as a significant predictor of users’ acceptance of new technologies, while some studies on smart clothing PIs have not considered ATTs.

Numerous studies have employed the TAM to investigate consumers’ BIs toward smart clothing, as summarized in Table 1. Some conclusions have been invalidated in other countries. Moreover, limited studies have examined the mediating role of PU and ATTs in the relationship between PEOU and PIs.

**Table 1:** Smart clothing purchase intention-related research.

Author	Foundation theories	Country/regions	Constructs	Key findings	Methodology
Bakhshian and Lee (2022) [23]	FEA, TRA	U.S.	FUN, expressive (EXP), AES, tracking (TCK), ATTs, SA, BIs	FUN→BI; EXP→ATT; EXP→BI; EXP→SA; AES→ATT; TCK→ATT; TCK→SA; TCK→BI; SA→ATT; SA→BI; ATT→BI.	CB-SEM
Wang and Wang (2021) [44]	FEA, TAM	——	FUN, AES, compatibility (COM), PU, PEOU, perceived performance risk (PR), ATTs, BIs	COM→PEOU; PEOU→PU; COM→PU; FUN→ATT; AES→ATT; PU→ATT; PEOU→ATT; FUN→BI; AES→BI; PU→BI; ATT→BI.	CB-SEM
Mahmood and Lee (2021) [18]	FEA, UTAUT	U.S.	FUN, EXP, AES, TCK, PE, effort expectancy (EE), SI, HC, privacy concern (PC), BIs	EXP→PE; EXP→EE; EXP→SI; TCK→PE; TCK→EE; SI→BI.	CB-SEM
Tsai et al. (2020) [28]	TAM	Taiwan	TA, perceived ubiquity (PB), RC, PU, PEOU, ATTs, BIs	PB→PU; PB→PEOU; PU→ATT.	PLS-SEM
Noh et al. (2016) [27]	TAM	Korea, China	Fashion innovation (FI), technology innovation (TI), PU, PEOU, perceived enjoyment (ENJ), perceived benefit, BIs	FI→PU; FI→ENJ; TI→PEOU; TI→ENJ; PEOU→PU; ENJ→PU; PU→BI.	CB-SEM
Hwang et al. (2016) [26]	FEA, TAM	U.S.	FUN, EXP, AES, PU, PEOU, PR, environmental concerns, ATTs, BIs	FUN→PU; FUN→PEOU; FUN→PR; COM→PU; COM→PEOU; COM→PR; AES→ATT; AES→BI; PEOU→PU; PU→ATT; PU→BI; PR→ATT; ATT→BI.	CB-SEM
Turhan (2013) [25]	TPB, TAM	Turkey	Self-efficacy (SE), fear of technological advances, cost, normative beliefs (NBs), PEOU, need compatibility, relative advantage, perceived behavioral control (PBC), subjective norms (SNs), PU, ATTs, BIs	PEOU→PU; ATT→BI.	CB-SEM
Chae (2010) [45]	TAM	Korea	TI, clothing involvement (CI), PU, PEOU, ATTs, BIs	TI→PU; CI→PU; CI→PEOU; PEOU→PU; PU→ATT; ATT→BI.	CB-SEM
Chae (2009) [46]	TAM	Korea	CI, PU, PEOU, ATT, BIs	PEOU→PU; PU→ATT.	CB-SEM

#### Perceived usefulness (PU)

PU indicates the extent to which people feel that adopting a new technology enhances their performance [38]. Previous studies have empirically supported the effect of PU on consumers’ adoption of wearable technology [42, 47]. According to Saleem et al. [48], PU has been developed to influence users’ ATTs and intentions toward e-shopping adoption. Thus, it is appropriate to investigate the influence of PU on consumers’ ATTs and intentions to purchase smart clothing. Since smart clothing can meet consumers’ unique needs in healthcare, work, and entertainment, it is important to understand its usefulness. Accordingly, the following hypotheses is proposed:

H1a: PU positively affects consumers’ ATTs.

H1b: PU positively affects smart clothing PIs.

#### Perceived ease of use (PEOU)

PEOU is highlighted as the degree to which individuals believe that adopting a new technology will be simple [38]. If consumers do not need to spend too much time or effort to master the wearing of smart clothing, smart clothing PIs will be enhanced. Several researchers have found a positive relationship between PEOU and PIs [49]. The effects of PEOU on PU and ATTs have been analyzed in various studies. According to Chuah et al. [50], PEOU positively and significantly impacts the PU of smartwatches as fashion accessories. Kasilingam [41] discovered that PEOU had a significant and positive impact on consumers’ ATTs toward c-commence chatbots. As PEOU is vital in the adoption of numerous information technology systems, it is logical to expect the same for smart clothing. Hence, the following hypotheses are suggested:

H2a: PEOU positively influences PU.

H2b: PEOU positively influences consumers’ ATTs.

H2c: PEOU positively influences consumers’ PIs.

#### Attitudes (ATTs)

Based on the TRA, which is a belief-attitude-behavioral intention model, ATTs dramatically influence consumers’ BIs. ATTs are defined as individuals’ inclinations and feelings or evaluative reactions to a subject. PIs are a personal behavioral tendency in terms of purchasing products or services. Furthermore, BIs can be influenced by ATTs in the TAM. Various empirical studies have demonstrated the influence of ATTs on PIs [51, 52]. In line with such studies, the following hypothesis is constructed:

H3: ATTs positively affect PIs.

#### Mediating role of PU and ATT

In a widely cited TAM paper, Davis hypothesized indirect effects of PU and PEOU on BIs through various mediators [38]. Specifically, PU was proposed to mediate the relationship between PEOU and PIs, with various studies across domains supporting this mediation [53]. Additionally, when consumers find smart clothing easy to wear, they are inclined to wear it more frequently. This ease of use elicits positive emotions, which in turn foster positive ATTs and enhance consumers’ PIs. Moreover, within smart clothing contexts, few studies have examined the sequential mediating roles of PU and ATTs in the relationship between PEOU and PIs. Therefore, the present study attempts to examine the indirect effects of PEOU on PI through the sequential mediators of PU and ATTs. The following hypotheses are proposed:

H4a: PU mediates the relationship between PEOU and PIs.

H4b: ATTs mediates the relationship between PEOU and PIs.

H4c: PU and ATTs sequentially mediate the positive relationship between PEOU and PIs.

Consistent with the original research by Davis [38], this study extends current research by examining ATTs as a mediator in the relationship between PU and PIs. If smart clothing provides more functions to meet consumers’ needs, consumers have a more favorable ATT toward purchasing smart clothing. Several studies have obtained similar conclusions in other contexts [54]. Hence, this study proposes the following hypothesis:

H5: ATT mediates the relationship between PU and PIs.

### External factors of the FEA model

The distinction between smart and ordinary clothing lies in the fact that smart clothing focuses on people’s actual needs [15]. Lamb and Kallal [21] proposed a user-centered model, namely, the FEA model, in which all three dimensions should be considered when identifying end-user clothing needs, and numerous studies have applied the FEA model in functional apparel and fashion design [55–57]. All related articles are presented in Table 1, revealing that the FEA model emerges as a commonly used model in research. Although the TAM is beneficial for explaining the BI to utilize new technologies, additional factors pertaining to the specific technology must be considered to fully comprehend its acceptance in specific contexts. Therefore, depending on the context of this research, this study applies the FEA model as the external variable to examine the influence on consumers’ intentions to purchase smart clothing.

#### Functionality (FUN) attribute

The FUN attribute refers to usability and usefulness related to wearable products performing their functions, which is crucial to consider when designing wearable products that are accepted by consumers [19]. In the context of clothing, FUN includes protection, thermal comfort, mobility, and safety, which are associated with clothing practicality and whether users accommodate the technology [44]. To meet the needs of consumers, smart clothing can currently implement various functions, such as positioning, alarms, physiological monitoring, and interaction. Various studies have indicated that the FUN attribute has a substantial impact on consumers’ use of smart clothing [26]. Therefore, this paper hypothesizes the following:

H6a: FUN positively influences PU.

H6b: FUN positively influences PEOU.

#### Expressiveness attribute

The EXP attribute relates to the communicative and symbolic aspects of clothing in social contexts [56]. In this research, EXP is defined as the degree to which the clothing aligns with an individual’s lifestyle, clothing, current needs, and social image [58]. This means not only the consumers’ present values but also how well they are consistent with the consumer’s current lifestyle. Consumers generally aim to minimize the effort needed to utilize innovative technology, while high compatibility tends to positively impact their willingness to adopt such technology. It has been suggested that the perceived EXP attribute positively influences users’ adoption of smart clothing [23]. As a result, if smart clothing aligns with the self-image that consumers wish to express, it may positively impact the factors related to technology acceptance. Thus, this paper hypothesizes the following:

H7a: EXP positively influences PU.

H7b: EXP positively influences PEOU.

#### Aesthetics (AES) attribute

The AES dimension refers to style and design, by which a product is supposed to look pleasing [21], and it includes novelty and beauty [55]. Creating AES when developing product appearance can improve usability, durability, elegant image, innovation, and positive user experience. According to Nam and Lee [19], design and consistency with the user’s image influence the acceptance of smart clothing. Sonderegger and Sauer [59] found that personalized design based on individuals’ aesthetic and formal preferences can greatly affect acceptance and use tendency. Moreover, the unique design of wearables enables users to immediately identify these devices [19]. In terms of smart clothing, the AES attribute is a critical element for the wearability and acceptability of the final product. Given these considerations, this paper proposes the following:

H8a: AES positively influences PU.

H8b: AES positively influences PEOU.

**Fig 1:**
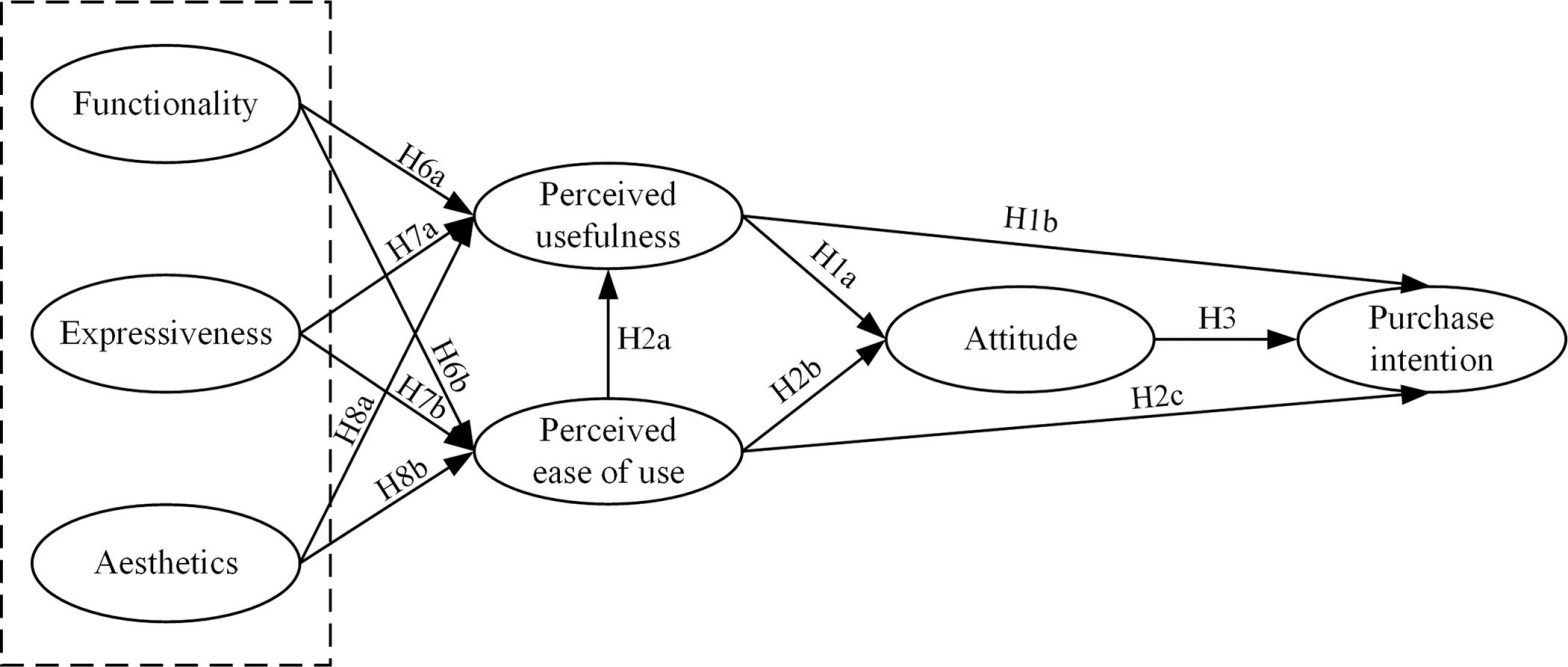
Theoretical model.

Based on the analysis above, this study constructs a theoretical model with the guidance of the FEA model and the TAM, as presented in Fig 1.

## Methodology

The Ethics Committee of Jiaxing University approved the ethical aspects of the “Understanding consumers’ intentions to purchase smart clothing using PLS-SEM and fsQCA” research plan, including the research informed consent, risk assessment, emergency plan. The research process underwent ongoing review for 12 months since approval.

All participants were protected and restricted by the Institutional Review Board (IRB). The Ethics Committee of Jiaxing University provided ethical approval for the study. Prior to data collection, all participants were informed about the purpose of the study, the benefits and risks associated with participation, and how their data would be used. All the tests were conducted after obtaining the participant’s consent, and the questionnaire was completed anonymously.

### Variables and measures

In the present research, a quantitative survey questionnaire was employed to measure each construct. The measurement of the variables comprises two parts. (1) The first part involves the measurement of variables associated with the theoretical model, which contains seven latent variables: FUN, EXP, AES, PU, PEOU, ATTs, and PIs. The items listed in Table 2 were evaluated using a 5-point Likert scale, ranging from 1 (strongly disagree) to 5 (strongly agree), and the items were modified from previously validated measures to suit the analysis. (2) The second part measures the sample demographics, including gender, age, education, and monthly disposable income. After minor revisions, a pilot study involving 35 respondents confirmed the survey’s accuracy. All measurement items, as depicted in Table 1, were extracted from established studies to ensure content validity.

**Table 2:** The constructs and measurement items.

Construct	Items	References
FUN	The comfort of smart clothing is critical.	[18, 26]
	The fit of smart clothing is critical.	
	The protection of smart clothing is critical.	
	Smart clothing is easy to wear and take off	
	Overall, I’m satisfied with the functionality of the smart garment.	
EXP	Smart clothing meets my needs.	[23, 26]
	Smart clothing coordinates well with other clothing I own.	
	Smart clothing fits well with my lifestyle.	
	Smart clothing will make me a leader in adopting new technologies.	
	Wearing smart clothing will impress others.	
AES	To me, the color of smart clothing is critical.	[18, 60]
	The texture of smart clothing is critical.	
	The design of smart clothing is critical.	
	Smart clothing is very fashionable.	
	Overall, I like the style of smart clothing.	
PU	Wearing smart clothing will improve my quality of life.	[38, 60]
	Wearing smart clothing will improve my work efficiency.	
	Smart clothing will meet my needs.	
	Wearing smart clothing can effectively improve my life.	
	Overall, smart clothing is very useful.	
PEOU	Wearing and using smart clothing do not require much thinking.	[26, 38, 60]
	The uses of smart clothing are clear and easy to understand.	
	Smart clothing can be easily used.	
	The interaction with smart clothing is quite straightforward and simple to comprehend.	
	Overall, I think smart clothing is easy to use.	
ATTs	I like the idea of using smart clothing.	[23, 26]
	It is wise to buy smart clothing.	
	Wearing smart clothing is an exciting experience.	
	Wearing smart clothing can be fun.	
	Overall, I have a positive attitude toward smart clothing.	
PIs	I will try smart clothing.	[60, 61]
	I’m interested in purchasing smart clothing when it is available for sale.	
	In the future, I intend to buy smart clothing.	
	I think it’s worth buying smart clothing.	
	I will recommend that others buy smart clothing.	

### Sample and data collection

In the scope of the current study, consumers who have bought and worn smart clothing are the target participants. Since smart clothing is an emerging technology, it was difficult to collect information about target consumers who wear such clothing. This research cooperated with a company that specializes in the design, production, and sale of smart clothing and commissioned it to collect consumer data. All participants were protected Institutional Review Board (IRB), which placed restrictions on their data. Prior to data collection, the purpose, benefits and risks of this research and the usage of data were explained to all participants. All tests were conducted after obtaining the participants’ consent, and the questionnaires were completed anonymously. The data were collected employing the snowball sampling method. To minimize sampling bias and errors, this study followed the guidelines outlined by Cohen and Arieli [62], implementing several measures as follows: (1) This article initiated parallel snowball networks that disseminated the electronic questionnaires across numerous WeChat customer groups linked to the company. This study specifically targeted individuals who had a comprehensive understanding of smart clothing, and it confirmed their smart clothing purchase history, to enhance the diversity in the sampling process. (2) This article combined snowball sampling with other methods, such as simple random sampling, to mitigate the subjectivity associated with sample selection in snowball sampling. (3) Additionally, this work set a limit on the number of times the questionnaire could be shared, permitting each participant to distribute the questionnaire only once. (4) Moreover, this research put restrictions on IP addresses, barring the same IP address from answering the survey questionnaire more than once. The data were collected between September and November 2022. A total of 236 subjects responded to the questionnaire. After removing missing data and duplicate answers, 225 usable responses were collected, yielding a 95.3% response rate. The final 225 responses exceed the sample size requirements of (1) having at least ten times the maximum quantity of formative indicators employed to measure a single construct, and (2) having at least ten times the largest number of structural paths pointing to any single latent construct within the structural model [63].

The demographic profile of the samples is presented in Table 3. Of the respondents, 183 were female and 42 were male. Regarding age, most respondents (86.2%) were 18–25, while only 31 respondents were older than 26. Regarding their educational level, most (97.3%) held a university degree or higher.

**Table 3:** The characteristics of the respondents.

Variable	Category	Frequency	Percentage
Gender	Female	183	81.3
	Male	42	18.7
Age	18–25	194	86.2
	26–30	21	9.3
	>31	10	4.4
Education	High school	6	2.7
	University	129	57.3
	Graduate degree or above	90	40.0
Disposable income	<CNY 1000	15	6.7
	CNY 1001–1500	80	35.6
	CNY 1501–2000	60	26.7
	CNY 2001–3000	34	15.1
	>CNY 3000	36	16.0

#### Common method bias

To mitigate any potential common method bias (CMB) arising from the self-report questionnaire, Harman’s single-factor test was employed [64]. If one factor explains over 50% of the total variance, the threat of CMB is significant [65]. The results indicate that a total of seven factors accounted for 71.23% of the total variance, with the first factor accounting for 40.07% of the total variance and no general factor being higher than the threshold of 50% [66]. In summary, there was minimal concern about CMB in this work. Meanwhile, multicollinearity was tested using variance inflation factors (VIFs). All independent variable VIFs ranged from 1.398 to 3.435, falling below the threshold value of 5.0 [30] and indicating no multicollinearity issues in the current study.

### Analytical approaches

Structural equation modeling (SEM) analyzes phenomenon-based structures using a confirmatory approach that accounts for measurement error and therefore provides more reliable conclusions about structural patterns for numerous indicators than other analysis methods, such as linear regression [67]. VB-SEM is chosen in this work for the following reasons. First, VB-SEM is a multivariate analytic method that possesses the capability to estimate causal models grounded in theoretical justifications [68]. Second, VB-SEM holds greater utility than CB-SEM in determining the relationship variance between dependent and independent variables [68]. Third, VB-SEM imposes a rather lenient limitation on the data distribution, which is more suitable for studies with nonnormal data and is quite robust to skewness [30]. Fourth, VB-SEM can perform estimation with smaller sample sizes and achieve greater statistical power than CB-SEM [69, 70]. Fifth, VB-SEM easily handles reflective, formative measurement models, and more complex models [71]. Finally, over the past decade, VB-SEM has been commonly applied in consumer behavior research, such as research on supply chain management [72], environment management [73], education management [74], hotel management [75], and marketing management [76]. Therefore, this study applied VB-SEM to perform data analysis. Following the Hair et al. guidelines [30], a two-step analytical procedure including measurement and structural models was evaluated using SmartPLS 3.0.

Unlike SEM’s reliance on linear association and a symmetric “net effect”, fsQCA employs an innovative configuration of causal antecedents to predict an outcome with either their presence or absence based on fuzzy set theory and fuzzy logic [77, 78]. fsQCA is a suitable approach for investigating complex complementarities and nonlinear relationships among constructs because it can be used to study a small number of cases that mix qualitative and quantitative methods. Additionally, configural analysis is used to investigate the configurational relationship between multiple solutions and the desired outcome [79]. The analysis was conducted using fsQCA 3.0 software. When using fsQCA software, the following three steps were incorporated into the modeling process. First, calibration converted the data into fuzzy sets by assigning values from 0 to 1. Second, necessary conditions identified determinants that could potentially influence achieving the desired outcomes. Third, a truth table algorithm was developed to report and interpret solutions.

## Results

This research combined PLS-SEM with fsQCA to conduct data analysis. This combination has been used in other areas [80, 81] and offers a deeper understanding of the antecedents in the smart clothing market context.

### PLS-SEM results

#### Reliability and validity analysis

PLS-SEM was applied to investigate the measurement model, including reliability, validity, and structural models. As indicated in Table 4, the Cronbach’s alpha values exceeded 0.7, and the composite reliability (CR) values ranged from 0.885 to 0.938, surpassing the recommended threshold of 0.7. These findings confirm high reliability and internal consistency. To assess convergent validity, the average variance extracted (AVE) was examined, it ranged from 0.578 to 0.751. The AVE values exceeded the cutoff value of 0.5, demonstrating adequate convergence. Moreover, the square root of the AVE for each construct, varying between 0.760 and 0.866, surpassed the corresponding correlation coefficients [82]. Additionally, all heterotrait monotrait ratio (HTMT) values were below 0.9 (see Table 5) [83], indicating excellent discriminant validity.

**Table 4:** Results of the reliability and validity of PLS-SEM.

Variables	Cronbach’s alpha	CR	AVE	FUN	EXP	AES	PU	PEOU	ATTs	PIs
FUN	0.840	0.885	0.607	**0.779**						
EXP	0.873	0.909	0.670	0.394	**0.818**					
AES	0.819	0.872	0.578	0.476	0.498	**0.760**				
PU	0.894	0.922	0.703	0.373	0.726	0.415	**0.838**			
PEOU	0.907	0.931	0.729	0.468	0.618	0.522	0.643	**0.854**		
ATTs	0.891	0.920	0.697	0.413	0.594	0.436	0.657	0.631	**0.835**	
PIs	0.917	0.938	0.751	0.283	0.485	0.319	0.574	0.496	0.716	**0.866**

Notes: The diagonal elements (in bold) represent the square root of the AVEs, while the off-diagonal elements show the correlations between variables.

**Table 5:** HTMT ratio of PLS-SEM.

Constructs	FUN	EXP	AES	PU	PEOU	ATTs	PIs
FUN							
EXP	0.444						
AES	0.589	0.570					
PU	0.411	0.818	0.468				
PEOU	0.520	0.693	0.601	0.712			
ATTs	0.469	0.671	0.504	0.728	0.697		
PIs	0.315	0.540	0.355	0.628	0.540	0.783	

#### Analysis of the structural model

**Fig 2:**
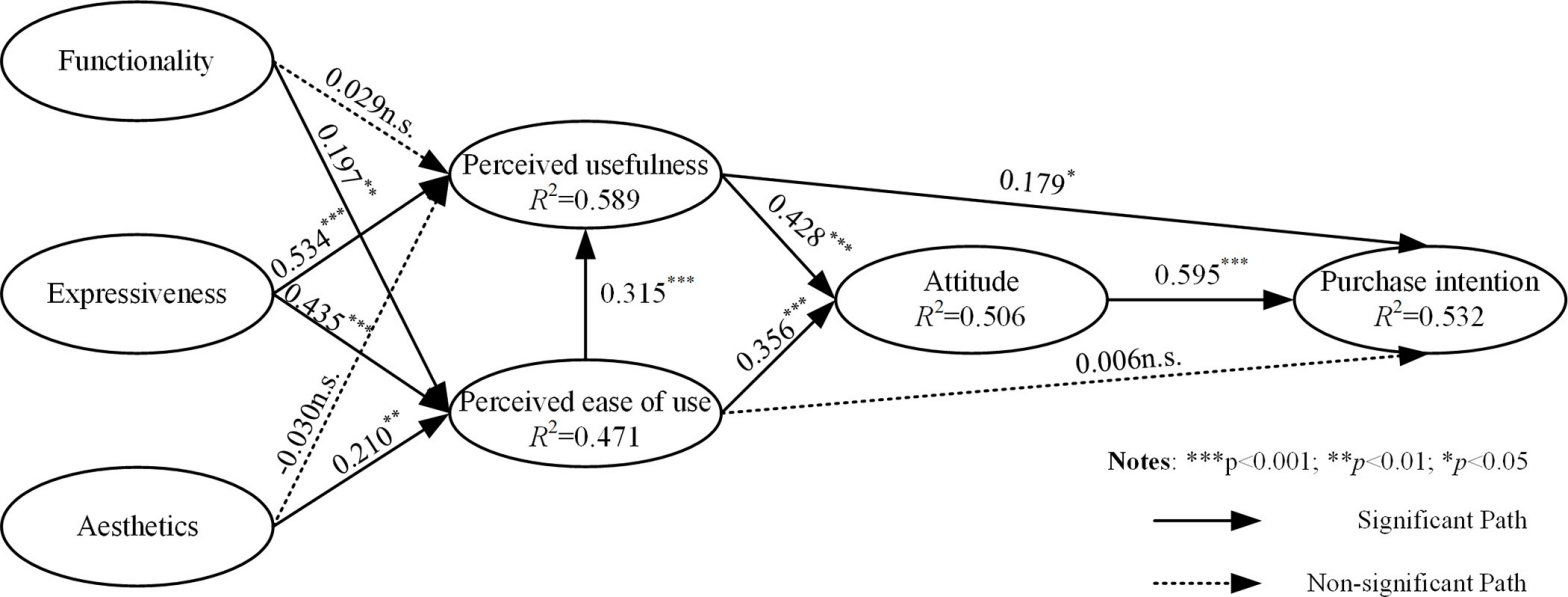
Path coefficient analysis.

This paper used the bootstrapping method with a simulation of 5000 random resamples to perform the path coefficient test. Table 6 represents the hypothesis test results of the model. Overall, 9 out of 11 proposed hypotheses were confirmed by the data, as presented in Fig 2, which presents the explained variance (*R*^2^) of the endogenous variables along with the path coefficients.

**Table 6:** Path results of the structural model.

Hypotheses	Paths	Coefficient (*β*)	*t* value	*p* value	Supported
H1a	PU -> ATTs	0.428	6.287	0.000	Yes
H1b	PU -> PIs	0.179	2.539	0.011	Yes
H2a	PEOU -> PU	0.315	4.787	0.000	Yes
H2b	PEOU -> ATTs	0.356	4.661	0.000	Yes
H2c	PEOU -> PIs	0.006	0.070	0.944	No
H3	ATT -> PI	0.595	7.179	0.000	Yes
H6a	FUN -> PU	0.029	0.538	0.591	No
H6b	FUN -> PEOU	0.197	3.183	0.001	Yes
H7a	EXP -> PU	0.534	9.366	0.000	Yes
H7b	EXP -> PEOU	0.435	7.542	0.000	Yes
H8a	AES -> PU	-0.030	0.537	0.591	No
H8b	AES -> PEOU	0.210	3.402	0.001	Yes

In the TAM, PU positively and significantly affected ATTs (β = 0.428, *p*<0.001) and PIs (β = 0.179, *p*<0.05), thereby confirming H1a and H1b. PEOU significantly influenced both PU (β = 0.315, *p*<0.001) and ATTs (β = 0.356, *p*<0.001), confirming H2a and H2b. However, the results show that PEOU did not affect PIs (β = 0.006, *p*>0.05), failing to support H2c. ATTs significantly influenced PIs (β = 0.595, *p*<0.001), supporting H3. Next, the examination of the FEA model confirmed the influence of FUN on PEOU (β = 0.197, *p* = 0.001), confirming H6b, whereas H6a (β = 0.029, *p* = 0.591) was not supported. This paper also observed that PU (β = 0.534, *p*<0.001) and PEOU (β = 0.435, *p*<0.001) were significantly affected by EXP, providing support for H7a and H7b. AES positively influenced PEOU (β = 0.210, *p* = 0.001); thus, H8b was supported. However, H8a (β = -0.030, *p* = 0.591) was not supported.

Importantly, path coefficients may not be quantified and evaluated until predictive power is assessed. To check the model fit, the standardized root mean square residual (SRMR), which represents the standardized difference between observed and predicted correlations [84], was utilized. The acceptable cutoff SRMR value for PLS path models is 0.08 [85]. For the PLS path model, the SRMR was 0.069, indicating a well-fitting model. Furthermore, the *R*^2^ value and predictive relevance (*Q*^2^) were used to evaluate the overall model quality. The structural model accounted for 53.2% of the variance in PIs, 50.6% of that in consumer ATTs, 47.1% of that in PEOU, and 58.9% of that in PU. These *R*^2^ values indicate a predictive accuracy of the model between moderate and strong [86]. Additionally, Stone–Geisser’s *Q*^2^ values were employed to assess predictive relevance, with values above zero indicating an accurate predictive capability of the model. A *Q*^2^ value of 0.391 was obtained, further validating the predictive applicability of the proposed model.

#### Mediation analyses

This research conducted a complementary mediation analysis following the approach of Zhao et al. (2010) [87]. This analysis was implemented with 95% bias-corrected bootstrap confidence intervals (CIs) with 5000 samples. As shown in Table 7, the direct effect of PEOU on PIs was nonsignificant, but the indirect effects were significant since the bias-corrected CIs excluded zero. Therefore, the results provide evidence that PU and ATTs serve as indirect-only mediators of the relationship between PEOU and PIs, implying that H4a, H4b and H4c were supported. This study employed a similar procedure to examine the relationship between PU and PIs. Both the direct and indirect effects proved significant, suggesting that ATTs serve as a complementary mediator in the relationship between PU and PIs; hence, H5 was supported.

**Table 7:** Mediation effect test.

**Panel A: H4**						
Hypotheses	β	*t* value	*p* value	Bias- corrected 95% CI		Decision
				Low	High	
Path estimate	0.006	0.070	0.944	-0.154	0.154	Not support
Mediation analysis						
H4a: PEOU -> PU -> PIs	0.056	2.235	0.025	0.073	0.214	Support
H4b: PEOU -> ATT -> PIs	0.212	4.119	0.000	0.119	0.321	Support
H4c: PEOU -> PU -> ATTs -> PIs	0.080	3.425	0.001	0.044	0.142	Support
Total indirect effects	0.348	6.366	0.000	0.250	0.468	Support
Total effect	0.354	4.514	0.000	0.186	0.492	Support
**Panel B: H5**						
Path estimate	0.179	0.071	2.539	0.042	0.318	Support
Mediation analysis						
H5: PU -> ATTs -> PIs	0.255	0.052	4.870	0.164	0.372	Support
Total effect	0.434	6.361	0.000	0.301	0.567	Support

### fsQCA results

#### Calibration

Since the variables were measured employing 5-point Likert scales, rescaling was required. Based on the suggestions made by Fiss [77], the full membership threshold was set to 5.0, the crossover point was set to 3.5, and full nonmembership was set to 1.0. Table 8 shows the results of this transformation and descriptive statistics.

**Table 8:** Calibrations and descriptive statistics.

Configurational element [Range]	Fuzzy set calibration			Descriptive statistics				
	Full membership	Crossover	Full non-membership	Mean	S. D	Min	Max	N-cases
FUN [1–5]	5.00	3.50	1.00	0.69	0.20	0.05	0.95	225
EXP [1–5]	5.00	3.50	1.00	0.55	0.23	0.05	0.95	225
AES [1–5]	5.00	3.50	1.00	0.67	0.20	0.05	0.95	225
PU [1–5]	5.00	3.50	1.00	0.61	0.22	0.05	0.95	225
PEOU [1–5]	5.00	3.50	1.00	0.61	0.22	0.05	0.95	225
ATTs [1–5]	5.00	3.50	1.00	0.65	0.20	0.05	0.95	225
PIs [1–5]	5.00	3.50	1.00	0.59	0.22	0.05	0.95	225

Consistent with the calibration process above, the outcome variable “PIs” was calibrated as “fs_PIs”, and the condition variables “FUN”, “EXP”, “AES”, “PU”, “PEOU” and “ATTs” were calibrated as “fs_FUN”, “fs_EXP”, “fs_AES”, “fs_PU”, “fs_PEOU”, and “fs_ATTs”, respectively.

#### Necessary conditions analysis

After the calibration, this research conducted a necessity analysis, examining whether any of the six factors is necessary for consumers’ smart clothing PIs. If the consistency score surpasses 0.90, the condition is deemed “necessary” [88]. As shown in Table 9, except for AES and PEOU, all other variables are necessary conditions for consumers’ smart clothing PIs. Importantly, analyzing necessary conditions is only one component of fsQCA, while examining sufficient causal combinations is essential.

**Table 9:** Necessity conditions.

Condition	Smart clothing purchase intention	
	Consistency	Coverage
fs_FUN	0.910	0.777
~ fs_FUN	0.464	0.886
fs_EXP	0.904	0.793
~ fs_EXP	0.491	0.886
fs_AES	0.825	0.884
~ fs_ AES	0.597	0.784
fs_PU	0.901	0.867
~ fs_PU	0.523	0.797
fs_PEOU	0.878	0.855
~ fs_PEOU	0.539	0.807
fs_ATTs	0.952	0.860
~ fs_ATTs	0.474	0.806

Note: “~” denotes the absence of a condition

#### Sufficient conditions analysis

The analysis of sufficient conditions was performed using a truth table of 2^*k*^ rows, where *k* denotes the number of outcome predictors, and each row denotes a combination of six predictors along with the frequency and consistency of each combination [32]. Frequency was defined as the number of observations for every combination, and because the sample of this study (225) was considered large (> 150 cases), a general suggested frequency threshold was a minimum of 3 [77]. Consistency was defined as the extent to which cases responded to the set-theoretic relationships denoted by a combination, with a cutoff point established at 0.80 [77]. Moreover, a minimum proportional reduction in inconsistency (PRI) should be taken into account [89]. Therefore, this work set the consistency and PRI consistency thresholds to 0.80 and 0.75, respectively. After calculating the consistency and coverage for all configurations, this study discovered that sufficient configurations with consistency and coverage exceed 0.8 and 0.2, respectively [36].

The standard analyses, produced by the fsQCA true table, reported complex, intermediate, and parsimonious solutions [32]. The research proposed intermediate and parsimonious solutions to distinguish between peripheral and core conditions [77]. Table 10 shows that each of the six causal combinations can lead to PIs, and that six equifinal configurations exist, with values ranging from 0.925 to 0.989. These configurations are as follows: Configuration 1: ~EXP * PU * ATTs; Configuration 2: FUN * AES * PU * ATTs; Configuration 3: FUN * PU * PEOU * ATTs; Configuration 4: AES * PU * PEOU * ATTs; Configuration 5: ~FUN * AES * ~EXP * PEOU * ATTs; and Configuration 6: FUN * AES * EXP * PEOU * ATTs. The consistency values were larger than 0.85, suggesting that all configurations were sufficient conditions leading to PIs. Furthermore, the overall solution coverage was 88.1%, indicating that a considerable share of the coverage was accounted for by the combinations related to consumers’ smart clothing purchase behaviors. Among them, solutions 2 and 3 accounted for 79.5% and 79.4% of cases leading to the outcome, respectively. The overall solution consistency was 0.915, suggesting that the six configurations could account for 91.5% of the cases with good explanatory power.

**Table 10:** Configurations for smart clothing purchase intentions.

Condition	1	2	3	4	5	6
FUN		•	•		Υ	•
EXP	Υ				Υ	●
AES		•		•	●	•
PU	●	●	●	●		
PEOU			•	•	●	•
ATTs	●	●	●	●	●	●
Consistency	0.959	0.925	0.934	0.937	0.989	0.947
Raw coverage	0.542	0.795	0.794	0.788	0.386	0.739
Unique coverage	0.016	0.013	0.021	0.017	0.007	0.012
Solution consistency	0.915					
Solution coverage	0.881					

Notes: Black circles (●) indicate the presence of a condition; crossed-out circles (ς) indicate its absence; large and small circles indicate core and peripheral conditions, respectively; blank spaces indicate that the condition may be either present or absent.

As demonstrated in Table 10, the core conditions specified in the various configurations were the presence of PU and ATTs. The findings indicated that consumers’ perception of usefulness and ATTs toward smart clothing were the most important significant conditions for fulfilling consumers’ strong desires to purchase smart clothing.

#### Predictive validity

**Fig 3:**
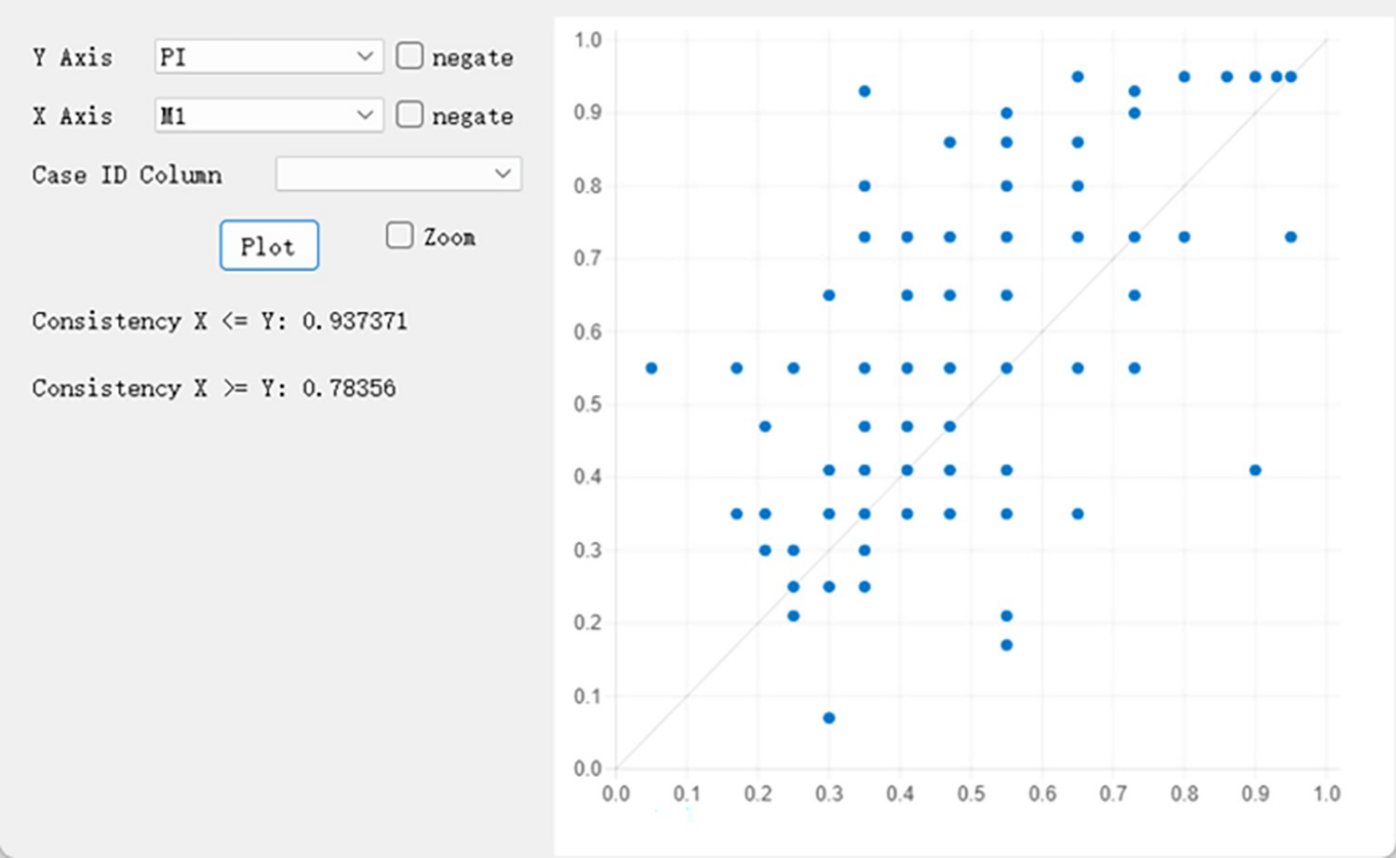
Fuzzy plot of model 1 (Table 11) using the holdout sample.

Validating the solutions for predictive validity is of utmost importance, as such validation assesses the extent to which the solutions accurately predict the values of the dependent variable across samples. Following the guidelines of Pappas and Woodside [36], the sample for this study was randomly divided into holdout samples and subsamples, identical analyses were conducted, and identical cutoff points were selected for both sets of samples, as described in the preceding sections. The solutions obtained from the subsample are presented in Table 11. Next, M1 (FUN*PU*PEOU*ATTs) was tested with the holdout sample data. Fig 3 indicates satisfactory consistency (0.937) and coverage (0.784), confirming the predictive validity of the proposed model. These findings indicate that the model has reliable and robust predictive ability for the values of the dependent variable.

**Table 11:** Predictive validity by using fsQCA.

Models from subsamples	Raw coverage	Unique coverage	consistency
M1: FUN*PU*PEOU*ATTs	0.8035	0.4796	0.9306
M2: ~FUN*AES*~EXP*~PU*PEOU*ATTs	0.3435	0.0195	0.9817
Solution coverage	0.8230		
Solution consistency	0.9305		

## Discussion and implications

### Discussion of the key findings

This study incorporates the FEA model, the TAM, and complexity theory to examine consumers’ smart clothing PIs. Additionally, the PLS-SEM and fsQCA approaches are applied to test these relationships.

Within the context of the consumers’ needs framework, the PLS-SEM results demonstrate that the FEA model is an antecedent of PU and PEOU, which indirectly influence smart clothing PIs. FUN is confirmed as positively influencing PEOU, consistent with previous findings [26]. However, its significant net effect on PU could not be identified. One possible explanation is that many consumers do not fully recognize the concept of smart clothing and still mistake smart clothing for functional clothing [2]. Moreover, smart clothing remains in the early stages of development, and some of the advertised functions do not meet consumer needs well. Additionally, EXP is one of the most significant factors of the FEA model that influences PU and PEOU. The significant net effect is aligned with that of previous studies, such as for smartwatches [90]. Furthermore, AES has a favorable net influence on PEOU, aligning with the findings of previous studies [91], but it has no significant net effect on PU. This reason may be that smart clothing is aesthetically unappealing and, thus, undesirable given the electrical components attached to or incorporated in the fabric.

Within the TAM framework, the PLS-SEM results show that PU and ATTs significantly affect consumers’ PIs. The significant net effects of PU and ATTs on shaping PIs align with previous studies, such as those examining electronic word of mouth (eWOM) [52] and online shopping [48], which emphasize the significance of consumers’ perceptions of usefulness and ATTs in achieving smart clothing PIs. Moreover, the findings show that PEOU has a nonsignificant effect on PIs, while PU and ATTs mediate the relationship between PEOU and PIs. The reason for this finding is that consumers might place more emphasis on PU than on how easy clothing is to use. If smart clothing is seen as providing significant benefits for consumers, PEOU might not play a substantial role in PIs. Additionally, consumers may appreciate the ease of smart clothing, and this appreciation may not transform into PIs unless it positively affects their ATTs toward smart clothing.

Although the PLS-SEM results offer insights into the net effects of antecedents on outcomes, fsQCA discloses several sufficient configurations of antecedent conditions. fsQCA reveals six key configurations of complexity theory that can drive high PIs (see the solutions in Table 10), and the results reaffirm that PU (consistency = 0.901) and ATTs (consistency = 0.952) are both necessary and sufficient conditions to achieve smart clothing PIs. In solutions 1 and 2, consumers who highly purchase smart clothing reported high FUN and AES needs or low EXP, as well as high PU and ATTs. Solutions 3 and 4 indicated that high FUN or AES needs, aligned with PU, PEOU, and ATTs, result in high PIs. Solution 5 indicated that high AES, PEOU, and ATTs, as well as low FUN and EXP, could produce high PIs. Conversely, solution 6 revealed that high levels of FUN, EXP, AES, PEOU and ATTs could also yield high PIs. The fsQCA results demonstrate that FUN and EXP are necessary for smart clothing PIs. Moreover, FUN is a condition in three of the six configurations (solutions 2, 3 and 6). Furthermore, EXP is a core condition in solution 6, while in solutions 1 and 5, the absence of EXP is a core condition, which is partially consistent with the PLS-SEM results (H7a and H7b). Although AES is not necessary, the presence of AES is a core condition in solution 5. Moreover, PU is the core condition in four of the six configurations (solutions 1, 2, 3, and 4) that lead to high smart clothing PIs, validating the PLS-SEM result (H1b). Additionally, ATTs are the core condition in all six configurations, and the result is congruent with H3.

The outcomes of the mixed-method analysis demonstrate that higher PU and ATTs could contribute to smart clothing PIs, supporting H1b and H3. Furthermore, this analysis reveals that PEOU significantly affects PU and ATTs, which is also consistent with similar findings on e-learning [92] and online food [93]. Solutions 3 and 4 indicate that PEOU, PU and ATTs can jointly lead to PIs, which supports the mediation hypotheses (H4a, H4b, and H4c). However, some results obtained from fsQCA contradicted those of PLS-SEM. For instance, FUN and AES had no impact on PU in the PLS-SEM analysis, while fsQCA confirmed the existence of several realities (i.e., solutions 2, 3, 4, and 6) in producing the same outcome. These findings were consistent with the equifinality principle, which states that more than one complicated configuration of antecedent conditions can produce the desired outcome. In accordance with the principle of causal asymmetry, fsQCA implied that the same antecedent within various solutions can have opposing impacts on smart clothing PIs, which depends on how they interact or combine with other attributes. Moreover, depending on the causal interactions, the absence or negation of some conditions might result in similar outcomes. For example, solution 5 suggested that low FUN and EXP can boost consumers’ smart clothing PIs, provided that the levels of AES, PEOU, and ATTs are high. PLS-SEM can confirm the predicted relationships between antecedents and PI outcomes but cannot provide such insights. Consequently, complexity theory demonstrated predictive validity for modeling consumers’ PIs toward smart clothing.

### Research implications

#### Theoretical implications

This work explores the relationships among FUN, EXP, AES, PU, PEOU, ATTs, and PIs, thus offering theoretical implications in two areas.

First, this research used a symmetric method, PLS-SEM, to investigate how various constructs are identified and grouped as determinants that predict smart clothing PIs. The findings also revealed that ATTs and PU are the most important and significant constructs. Moreover, this research explored the underlying mechanisms of the mediation process. The mediation test illustrated that PU and PEOU have indirect effects on PIs through ATTs. Additionally, prior research has extensively identified the positive influences of PU and ATTs on PIs, while few studies have examined the effect of PEOU on PIs within the specific context of smart clothing. Through mediation analysis, it can be concluded that PU and ATTs act as indirect-only mediators between PEOU and PIs.

Second, in contrast to previous studies that primarily employed symmetrical modeling (multiple regression, PLS-SEM) to examine the net effect of each isolated antecedent on consumers’ intentions to purchase smart clothing, this research adopted a configurational approach rooted in complexity theory. By employing fsQCA, a more comprehensive and nuanced understanding of the phenomenon of consumers’ PIs toward smart clothing was achieved. Unlike symmetric techniques, which fail to identify the intricate causal conditions necessary for achieving the desired outcome, fsQCA enabled a more extensive exploration of the complex interplay between constructs. By applying fsQCA, six distinct models were developed to elucidate how configurations of constructs influence users’ behavior in the smart clothing context. Moreover, the fsQCA findings demonstrate alignment with complexity theory principles in the smart clothing context. Hybrid approaches integrating PLS-SEM and fsQCA empower researchers to delve into the complexities of consumer behavior in greater depth and detail.

#### Managerial implications

The rapid growth of smart clothing, an emerging technology recognized as a disruptive innovation in the garment industry, necessitates a strategic approach for businesses in this sector, especially in the context of markets such as China. This research provides valuable insights and important managerial implications for manufacturers and marketers in this innovative sphere.

First, within the framework of consumers’ needs, this research highlights that FUN, EXP, and AES greatly influence PEOU. Additionally, EXP has a great impact on PU. For instance, Yang et al. [94] noted that smart clothing that is washable, stretchable, and flexible is more acceptable to users and could meet the needs of society. The FUN of smart clothing can be enhanced through modular designs, allowing products to serve multiple purposes based on consumers’ needs. By leveraging the power of the IoT, big data, cloud computing, mobile computing, and social media, smart clothing can provide a variety of intelligent features, such as alarms, positioning, physiological monitoring and interaction, and entertainment. In fact, the complex and sophisticated functions of smart clothing may not improve quality of life but bring much inconvenience, causing consumers to lose interest in smart clothing over time [15]. Fashion designers need to listen to consumers and integrate the latest fashionable elements and technical attributes into the design of smart clothing to make it innovative and aesthetically pleasing clothing, and consumers could participate in smart clothing design [44, 95]. Additionally, special attention should be paid to the needs of specific groups, such as elderly individuals. Through style and structural design, smart clothing can be made easy to wear. It is crucial to remember that smart clothing needs to strike a balance between the FUN and AES requirements of consumers to appeal to the broadest consumer group, thus increasing market acceptability.

Second, this study confirms that PU and ATTs are critical factors in smart clothing PIs and that the effect of PEOU on PI is mediated by PU and ATTs. These results suggest the need for manufacturers to focus on conveying the benefits of their smart clothing products clearly and fostering a positive brand image. Doing so can be achieved through comprehensive marketing strategies such as detailed product demonstrations, user testimonials, and educational campaigns. Notably, companies should also ensure that their products are user-friendly, featuring intuitive user interfaces, clear instructions, and robust customer support. Providing effective solutions to potential issues such as smart clothing washing and maintenance can also enhance PEOU. As demonstrated, ATTs play a critical role in predicting smart clothing PIs. Manufacturers and marketers should concentrate on measures to improve individuals’ ATTs toward this innovative technology. For smooth entry into the market, businesses should emphasize the user experience and engage in diverse marketing tools such as social media marketing [96] and key opinion leader (KOL) marketing [97].

## Conclusions, limitations and future research

### Conclusions

This paper draws on the FEA model, the TAM and complexity theory to establish a theoretical model for understanding the formation of consumers’ smart clothing purchase behaviors. The study empirically analyzes survey-based data using two complementary analytical approaches, variance-based PLS-SEM and case-oriented fsQCA, providing a comprehensive and in-depth research perspective for understanding the formation mechanism of smart clothing purchase behavior in China.

The net effects of each antecedent on PIs are analyzed through PLS-SEM. The PLS-SEM results demonstrate that all FEA dimensions influence PEOU, while the EXP attribute does not significantly impact PU. Additionally, all direct relationships within the TAM are significant, except for the link between PEOU and PIs. Furthermore, PU and ATTs exhibit major mediating roles across the proposed relationships.

Regarding the high levels of complexity theory, this theory is successfully extended to the smart clothing context. The fsQCA approach delivers granular insight into how antecedents interact to influence consumers’ smart clothing PIs, deriving six different configurations of variables for achieving high levels of smart clothing PIs, with the combination of FUN, AES, PU, and ATTs being the best solution.

In summary, this research enriches the theoretical understanding of the complex causality and interactions explaining smart clothing PIs. The findings of this research offer valuable guidance for enterprises and marketers in formulating and implementing marketing strategies to encourage consumers’ smart clothing purchase behavior.

### Limitations and future research

Several limitations to this research suggest meaningful future research directions. First, given that this study was conducted in China, its results may not precisely align with purchase behaviors in other countries, due to significant cultural, economic, and ethnic differences. Future research should validate the applicability of the model in both developing and developed countries with varied cultural contexts. Second, this study examined only the effect of the influencing factors of smart clothing products themselves and technology acceptance on PIs. Future works should consider additional constructs, such as self-efficacy (SE), price and innovativeness, to explore the influence of multiple factors on smart clothing PIs. Finally, this work assessed the determinants of smart clothing PIs by drawing on the FEA model and the TAM. Future studies should investigate antecedents from other theoretical lenses, such as innovation diffusion theory (IDT).

## Supporting information

S1 File (CSV)
